# Liver function predicts survival in patients undergoing extracorporeal membrane oxygenation following cardiovascular surgery

**DOI:** 10.1186/s13054-016-1242-4

**Published:** 2016-03-11

**Authors:** Christian Roth, Lore Schrutka, Christina Binder, Lukas Kriechbaumer, Gottfried Heinz, Irene M. Lang, Gerald Maurer, Herbert Koinig, Barbara Steinlechner, Alexander Niessner, Klaus Distelmaier, Georg Goliasch

**Affiliations:** Department of Internal Medicine II, Medical University of Vienna, Vienna, Austria; University Clinic of Orthopedics, Paracelsus Medical University Salzburg, Salzburg, Austria; Department of Anesthesia and Intensive Care Medicine, Karl Landsteiner University of Health Sciences, University Hospital Krems, Krems an der Donau, Austria; Division of Cardiothoracic and Vascular Anesthesia and Intensive Care Medicine, Medical University of Vienna, Vienna, Austria

**Keywords:** Extracorporeal membrane oxygenation, Liver function, Bilirubin, Alkaline phosphatase, Cardiovascular surgery, Outcome, Mortality

## Abstract

**Background:**

Extracorporeal membrane oxygenation (ECMO) represents a valuable and rapidly evolving therapeutic option in patients with severe heart or lung failure following cardiovascular surgery. However, despite significant advances in ECMO techniques and management, prognosis remains poor and accurate risk stratification challenging. We therefore evaluated the predictive value of liver function variables on all-cause mortality in patients undergoing venoarterial ECMO support after cardiovascular surgery.

**Methods:**

We included into our single-center registry a total of 240 patients undergoing venoarterial ECMO therapy following cardiovascular surgery at a university-affiliated tertiary care center.

**Results:**

The median follow-up was 37 months (interquartile range 19–67 months), and a total of 156 patients (65 %) died. Alkaline phosphatase and total bilirubin were the strongest predictors for 30-day mortality, with adjusted hazard ratios (HRs) per 1–standard deviation increase of 1.36 (95 % confidence interval [CI] 1.10–1.68; *P* = 0.004) and 1.22 (95 % CI 1.07–1.40; *P* = 0.004), respectively. The observed associations persisted for long-term mortality, with adjusted HRs of 1.27 (95 % CI 1.03–1.56; *P* = 0.023) for alkaline phosphatase and 1.22 (95 % CI 1.07–1.39; *P* = 0.003) for total bilirubin.

**Conclusions:**

The present study demonstrates that elevated values of alkaline phosphatase and total bilirubin are sensitive parameters for predicting the short-term and long-term outcomes of ECMO patients.

**Electronic supplementary material:**

The online version of this article (doi:10.1186/s13054-016-1242-4) contains supplementary material, which is available to authorized users.

## Background

Venoarterial extracorporeal membrane oxygenation (ECMO) is a lifesaving rescue therapy in patients with refractory heart and lung failure after cardiovascular surgery. However, despite significant advances in ECMO techniques and management over the last several years [1], prognosis remains poor [2, 3]. The underlying causes of death in this highly vulnerable patient population vary from cardiovascular events to organ failure, including gastrointestinal, hepatic, renal, neurologic, coagulation, endocrine, or immunologic system failure [4–7]. This diversity of potential complications challenges an accurate prediction of clinical outcome. The identification of novel risk parameters might significantly improve risk assessment before ECMO implantation and facilitate early recognition of organs prone to failure.

Generally, early hepatic dysfunction represents an independent risk factor for poor prognosis in critical ill patients, especially in those with heart failure [8–11]. Even a subtle elevation of liver variables such as bilirubin, alanine aminotransferase, alkaline phosphatase, and γ-glutamyl transferase was found associated with clinical outcome in acutely ill patients [12]. An extended intensive care unit (ICU) stay and higher mortality rates were observed in those patients with elevated liver variables [8, 11]. Derangements of liver variables in this population are caused mainly by hepatic ischemia, sepsis, or iatrogenic pharmacological effects [13–15].

Patients with ECMO support are in a compromised hemodynamic condition and therefore vulnerable to ischemic events. Liver failure and bowel ischemia are common and severe complications in patients undergoing ECMO support [4–6, 16]. Whether preoperative liver variables may predict mortality in patients undergoing ECMO support following cardiovascular surgery has not been investigated yet. We therefore analyzed the predictive value of liver function variables on all-cause mortality in patients undergoing venoarterial ECMO support after cardiovascular surgery.

## Methods

### Study population

We enrolled patients undergoing veno-arterial ECMO support following cardiovascular surgery between September 2003 and June 2014 into our observational single-center registry. All subjects were included at the Vienna General Hospital, a university-affiliated tertiary center, as previously described [17]. All patients admitted to the Medical University of Vienna provided written consent at hospital admission. The study is in line with the Declaration of Helsinki and was approved by the ethics committee of the Medical University of Vienna.

### Study endpoints and clinical definitions

Liver function parameters are part of the routinely measured blood values at the time of admission at the Vienna General Hospital, and they are measured according to local laboratory standard procedures. Liver function parameters included the following measurements: alkaline phosphatase (U/L), total bilirubin (mg/dl), aspartate transaminase (U/L), alanine transaminase (U/L), γ-glutamyl transferase (U/L), albumin (g/L), and Normotest (Nycomed, Oslo, Norway) (%). At the time of ICU admission, the Simplified Acute Physiology Score (SAPS)-3 [18] and the Sequential Organ Failure Assessment (SOFA) score [19] were recorded. Additionally, the De Ritis ratio (aspartate transaminase/alanine transaminase [AST/ALT]) and the European System for Cardiac Operative Risk Evaluation (EuroSCORE) were calculated [20]. Hypoxic hepatitis was defined as a dramatic but transient increase in serum aminotransferase activity reaching at least 20 times the upper limit of normal within 72 h following ECMO implantation [21]. The glomerular filtration rate was estimated using the Modification of Diet in Renal Disease formula [13]. All-cause 30-day mortality was defined as primary and long-term mortality during the whole observation period as a secondary study endpoint. Mortality data were obtained by screening the national register of death.

### ECMO management

An indication for ECMO support was given in patients with clinical signs of cardiogenic shock such as systolic arterial hypotension (<80 mmHg) and anaerobic metabolism (i.e., elevated serum lactate levels), signs of end organ failure, and metabolic acidosis despite optimized supportive measures (i.e., inotropes, fluids, intraaortic balloon pump). The ECMO circuit comprised a centrifugal pump console (Bio-Console 560 Speed Controller System from Medtronic, Minneapolis, MN, USA; or CARDIOHELP system; Maquet, Rastatt, Germany) and a membrane oxygenator (Affinity-NT Oxygenation System, Medtronic; or HLS Module Advanced, Maquet). The ECMO system was serviced by an experienced perfusionist as well as the on-shift intensive care physician. All components of the ECMO system were coated with heparin. The fraction of inspired oxygen on the oxygenator was adjusted to maintain a target partial pressure of oxygen of 80–100 mmHg. If clots or significant fibrin depositions were present or if blood oxygenation declined drastically, the ECMO circuit was changed. Under ECMO support, ventilation was reduced with peak airway pressures below 25 cmH_2_O and respiratory tidal volumes between 6 and 8 ml/kg.

### Statistical methods

Discrete data were presented as count and percentage and analyzed by using a χ^2^ test. Continuous data were presented as median and interquartile range (IQR) and compared using the Kruskal-Wallis test. Cox proportional hazards regression analysis was applied to assess the effect of the respective liver function parameter on survival. Results were expressed as the hazard ratio (HR) for a 1–standard deviation (SD) change with the respective 95 % confidence intervals (95 % CIs). To account for potential confounding effects, we adjusted for established risk factors, including age, sex, SAPS-3 score, left ventricular function, hypertension, diabetes, estimated glomerular filtration rate (eGFR), type of cardiovascular surgery, and year of study enrollment. Interactions between liver function parameters and all variables included in the multivariable model were tested by entering interaction terms in the Cox proportional hazards regression models. The discriminatory power of the respective variables was assessed using Harrell’s c-statistic. Two-sided *P* values less than 0.05 were used to indicate statistical significance. All analyses were done using STATA 11 software (StataCorp, College Station, TX, USA) and IBM SPSS 22.0 software (IBM, Armonk, NY, USA).

## Results

### Baseline characteristics

We included a total of 240 patients with a median age of 65 years (IQR 55–72) who underwent ECMO support after cardiovascular surgery. One hundred seventy-two patients (72 %) were male. The median SAPS-3 score, median EuroSCORE, and median SOFA score of the study population were 43 (IQR 36–51), 10 (8–13), and 12 (10–14), respectively. Hypoxic hepatitis was diagnosed in 35 patients (15 %) within 72 h following ECMO implantation. Indications for ECMO implantation were weaning failure from cardiopulmonary bypass (60 %), postoperative cardiogenic shock (20 %), immediate posttransplant cardiac graft failure (6 %), postoperative respiratory failure (4 %), postoperative bleeding and/or tamponade with cardiogenic shock (4 %), and miscellaneous conditions (6 %). In 44 % of patients, ECMO implantation was performed femoral-femoral, in 46 % of patients it was femoral-subclavian, and in 10 % of patients it was femoral-central. ECMO support was required in 59 patients after valve surgery, in 24 after coronary artery bypass graft (CABG) surgery, in 56 after combined CABG-valve surgery, in 51 patients after cardiac transplantation, in 21 patients after ventricular assist device implantation, in 17 after aortic reconstruction, and in 12 after other cardiovascular surgeries. Detailed information on baseline characteristics are given in Table 1. Detailed baseline characteristics for the study population by type of cardiovascular surgery and by time of study enrollment are given in Additional file 1: Tables S1 and S2.

**Table 1:** Baseline characteristics of total ECMO study population (*n* = 240)

Characteristics	Total study population (*n* = 240)
Baseline characteristics at hospital admission	
Age, years, median (IQR)	65 (55–72)
Male sex, *n* (%)	172 (72)
EuroSCORE (additive), points (IQR)	10 (8–13)
Procedure duration, h:min (IQR)	8:00 (6:08–9:37)
Hypertension, *n* (%)	169 (70)
Diabetes, *n* (%)	66 (28)
Hypercholesterolemia, *n* (%)	125 (52)
Coronary artery disease, *n* (%)	124 (52)
Left ventricular ejection fraction	
30–44 %, *n* (%)	35 (15)
< 30 %, *n* (%)	94 (39)
Creatinine, mg/dl (IQR)	1.3 (1.1–1.8)
eGFR, ml/min/1.73 m^2^ (IQR)	51.3 (38.9–68.5)
Blood urea nitrogen, mg/dl (IQR)	24.2 (18.2–36.1)
Cholesterol, mg/dl (IQR)	143 (104–182)
C-reactive protein, mg/dl (IQR)	1.0 (0.3–4.3)
White blood cells, g/L (IQR)	8.0 (6.1–11.1)
Thrombocytes, G/L (IQR)	188 (134–241)
Liver parameters	
Bilirubin total, mg/dl (IQR)	1.1 (0.6–1.6)
Alkaline phosphatase, U/L (IQR)	81 (61–110)
Aspartate transaminase, U/L (IQR)	33 (23–67)
Alanine transaminase, U/L (IQR)	27 (18–47)
AST/ALT ratio, *n* (IQR)	1.3 (0.9–2.1)
γ-Glutamyl transferase, U/L (IQR)	54 (32–105)
Albumin, g/L (IQR)	38.6 (30.8–42.4)
Normotest, % (IQR)	75 (50–94)
Post-ECMO implantation (first 24 h)	
SAPS-3, *n* (%)	43 (36–51)
SOFA score, *n* (IQR)	12 (10–14)
ECMO flow, L/min (IQR)	3.36 (2.50–4.25)
ECMO rotation, rpm (IQR)	3000 (2485–3500)
ECMO gas flow, L/minute (IQR)	2.5 (2.0 − 3.0)
ECMO FiO_2_, % (IQR)	70 (60–100)
ECMO duration, days, median (IQR)	4 (3–7)
Hemodynamic parameters (at ICU admission)	
Mean arterial pressure, mmHg (IQR)	72 (65–79)
Cardiac output, L/minute	3.9 (2.8–5.1)
ScVO_2_, % (IQR)	71 (64–77)
Central venous pressure, mmHg (IQR)	14 (11–16)
Medication (first 24 h post-ECMO)	
Noradrenaline, *n* (%)	234 (98)
Noradrenaline (maximum dose), μg/kg/minute	0.28 (0.13–0.58)
Dobutamine, *n* (%)	218 (91)
Dobutamine (maximum dose), μg/kg/minute	4.78 (2.88–7.14)
Vasopressin, *n* (%)	85 (35)
Vasopressin (maximum dose), U/h (IQR)	3.0 (2.0–4.0)

*ALT* alanine aminotransferase, *AST* aspartate aminotransferase, *ECMO* extracorporeal membrane oxygenation, *eGFR* estimated glomerular filtration rate, *EuroSCORE* European System for Cardiac Operative Risk Evaluation, *FiO*_*2*_ fraction of inspired oxygen, *ICU* intensive care unit, *IQR* interquartile range, *SAPS* Simplified Acute Physiology Score, *ScVO*_*2*_ central venous oxygen saturation, *SOFA* Sequential Organ Failure Assessment

### Liver function parameters and outcomes

**Fig. 1:**
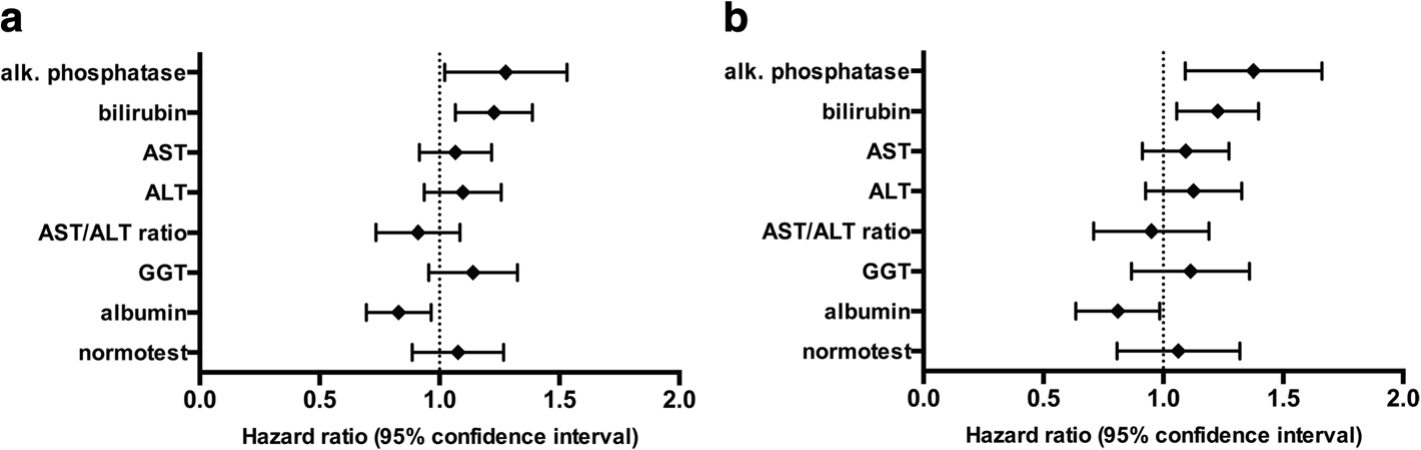
Forest plot displaying the multivariate Cox regression results of the respective liver function parameters for 30-day mortality (**a**) and long-term mortality (**b**). Hazard ratios (HR) refer to a 1-SD increase/decrease in continuous variables

The median follow-up was 37 months (IQR 19–67 months). During this period, 65 % of patients (*n* = 156) died. Alkaline phosphatase and total bilirubin were the strongest predictors of outcome among liver function parameters in the univariable Cox regression analysis. Serum levels of alkaline phosphatase at hospital admission displayed a direct association with 30-day mortality, with a crude HR per 1-SD of 1.23 (95 % CI 1.02–1.50; *P* = 0.035) for 30-day mortality. Comparable results were obtained for total bilirubin, with a crude HR per 1 SD of 1.16 (95 % CI 1.02–1.32; *P* = 0.028) for 30-day mortality, as well as for long-term mortality, with a HR per 1 SD of 1.15 (95 % CI 1.02–1.30; *P* = 0.028). AST, ALT, AST/ALT ratio, γ-glutamyl transferase, and Normotest showed no association with clinical outcome. Detailed results of the univariable Cox regression analysis for liver function parameters are given in Table 2. The discriminatory power measured by Harrell’s c-statistic of total alkaline phosphate was 0.53 for 30-day mortality and 0.51 for long-term mortality, whereas total bilirubin yielded c-statistics of 0.56 for 30-day mortality and 0.57 for long-term mortality. These effects were even more pronounced after adjustment for potential confounders for alkaline phosphatase, with adjusted HRs of 1.36 (95 % CI 1.10–1.68; *P* = 0.004) for 30-day mortality and 1.27 (95 % CI 1.03–1.56; *P* = 0.023) for long-term mortality, as well as for total bilirubin with adjusted HRs 1.22 (95 % CI 1.07–1.40; *P* = 0.004) for 30-day mortality and 1.22 (95 % CI 1.07–1.39; *P* = 0.003) for long-term mortality (Table 3). Forest plot displaying the multivariate Cox regression results of the respective liver function parameters for mortality is depicted in Fig. 1. Furthermore, we observed a significant association between the occurrence of hypoxic hepatitis and 30-day mortality (crude HR 3.47, 95 % CI 2.15–5.61, *P* < 0.001; adjusted HR 4.02, 95 % CI 2.35–6.89) and long-term mortality (crude HR 2.85, 95 % CI 1.91–4.25, *P* < 0.001; adjusted HR 3.5, 95 % CI 2.08–5.09, *P* < 0.001). There was no association between baseline levels of the presented liver parameters and the occurrence of hypoxic hepatitis (data not shown). We did not observe any significant interactions between liver function and the variables included in the multivariate model (data not shown).

**Table 2:** Univariable Cox proportional hazards model of liver parameters before ECMO implantation

		30-day mortality		Long-term mortality	
Baseline parameters	SD	HR (95 % CI)	*P* value	HR (95 % CI)	*P* value
Alkaline phosphatase, U/L	68.9	1.23 (1.02–1.50)	0.035	1.14 (0.95–1.38)	0.16
Bilirubin total, mg/dl	2.0	1.16 (1.02–1.32)	0.028	1.15 (1.02–1.30)	0.028
Aspartate transaminase, U/L	303.5	1.08 (0.92–1.27)	0.36	1.06 (0.91–1.23)	0.45
Alanine transaminase, U/L	192.0	1.07 (0.90–1.27)	0.45	1.04 (0.90–1.21)	0.60
AST/ALT ratio, *n*	1.9	0.97 (0.78–1.21)	0.81	0.95 (0.80–1.12)	0.51
γ-Glutamyl transferase, U/L	133.3	1.07 (0.87–1.32)	0.54	1.10 (0.94–1.29)	0.22
Albumin, g/L	8.7	0.83 (0.68–1.01)	0.06	0.86 (0.74–1.00)	0.049
Normotest, %	30.0	1.02 (0.82–1.26)	0.87	1.06 (0.90–1.24)	0.50

*ALT* alanine aminotransferase, *AST* aspartate aminotransferase, *CI* confidence interval, *HR* hazard ratio, *SD*, standard deviation

Hazard ratios refer to a 1-SD increase and/or decrease in continuous variables

**Table 3:** Adjusted Cox proportional hazard model of liver parameters beforre ECMO implantation

		30-day mortality		Long-term mortality	
Baseline parameters	SD	Adjusted HR (95 % CI)	*P* value	Adjusted HR (95 % CI)	*P* value
Alkaline phosphatase, U/L	68.9	1.36 (1.10–1.68)	0.004	1.27 (1.03–1.56)	0.023
Bilirubin total, mg/dl	2.0	1.22 (1.07–1.40)	0.004	1.22 (1.07–1.39)	0.003
Aspartate transaminase, U/L	303.5	1.07 (0.90–1.27)	0.44	1.05 (0.91–1.22)	0.48
Alanine transaminase, U/L	192.0	1.11 (0.93–1.33)	0.27	1.08 (0.93–1.26)	0.30
AST/ALT ratio, *n*	1.9	0.90 (0.70–1.17)	0.43	0.89 (0.74–1.08)	0.24
γ-Glutamyl transferase, U/L	133.3	1.09 (0.87–1.35)	0.47	1.12 (0.95–1.31)	0.18
Albumin, g/L	8.7	0.81 (0.65–1.01)	0.06	0.83 (0.71–0.98)	0.028
Normotest, %	30.0	1.03 (0.81–1.31)	0.81	1.06 (0.88–1.26)	0.56

*ALT* alanine aminotransferase, *AST* aspartate aminotransferase, *CI* confidence interval, *HR* hazard ratio, *SD*, standard deviation

Hazard ratios refer to a 1-SD increase and/or decrease in continuous variables. HRs are adjusted for all variables in the clinical confounder model (i.e., for age, sex, Simplified Acute Physiology Score-3, left ventricular function, hypertension, diabetes, estimated glomerular filtration rate, type of cardiovascular surgery, and year of study enrollment.

## Discussion

The present study highlights the impact of preoperative liver function on clinical outcome in patients undergoing venoarterial ECMO support following cardiovascular surgery. Alkaline phosphatase and total bilirubin were identified as the strongest predictors of 30-day and long-term mortality. Additionally, low albumin levels were found to be associated with poor long-term survival. After adjustment for potential confounders, these associations were even more pronounced.

The high mortality rate in cardiovascular surgery patients requiring ECMO therapy emphasizes the importance of optimized risk stratification before ECMO implantation [7, 22]. The identification of novel risk factors might help to select appropriate patients for ECMO therapy. Liver failure is a common and significant complication in this patient population [4–6, 16]. In general, derangements of hepatic liver variables represent established risk factors for adverse outcome in critically ill patients [8–11]. Particularly in surgical patients, the hepatic system has a significant impact on clinical outcome [23, 24]. Consequently, bilirubin, a frequently used marker to estimate liver function, has been incorporated into established ICU critical illness scoring systems such as SAPS [25] and SOFA [19]. A comprehensive study investigating the impact of the preoperative hepatic biochemical profile on clinical outcome in ECMO patients has not been done before. We identified alkaline phosphatase and total bilirubin as the strongest predictors of clinical outcome in this highly vulnerable study population of cardiovascular surgery patients. This finding underlines the mutual relationship between heart and liver that has been spotlighted by both hepatologists and cardiologists over the last several years [26].

Generally, elevation of alkaline phosphatase and bilirubin is considered as a cholestatic pattern. Cholestasis is defined as a decline of bile flow into the duodenum that may be caused by impaired secretion by hepatocytes or obstruction through intra- or extrahepatic bile ducts. Particularly, adverse effects of medications and systemic sepsis may be the underlying mechanism for elevated cholestatic parameters in the present study cohort. As preoperative bilirubin and alkaline phosphatase levels were in the upper normal range or only slightly elevated in the present study population, significant cholestasis is not suggested to be the sole organic correlate for poor outcome. The commonality of our study population is the severe cardiovascular disease that may itself affect liver function. The pathophysiology of cardiac hepatopathy is, on one hand, impaired arterial perfusion caused by acute heart failure and, on the other hand, passive congestion resulting from an increased central venous pressure due to elevated right ventricular pressure and right-sided heart failure [26]. Mostly, forward and backward failure coexist and aggravate each other in patients with severe heart failure [13]. Furthermore, hepatic derangements are not necessarily caused by primary liver disease, but may be a consequence of severe illness and may represent a surrogate marker of the generalized stress response [12, 27].

In line with our results, elevated levels of alkaline phosphatase or bilirubin were identified to predict all-cause mortality, cardiovascular death, or hospitalization for heart failure in patients with advanced heart failure [28, 29]. Hemodynamic alterations triggered by heart failure were found to be associated with the development of hypoxic hepatitis [30, 31]. Hypoxic hepatitis is a form of hepatic injury following acute arterial hypoxemia, hypoperfusion with consecutive ischemia, and passive congestion of the liver that significantly affects prognosis and the outcome of critically ill patients [32]. It is tempting to speculate that preoperative determination of alkaline phosphatase and bilirubin levels can detect patients with early-stage hepatocardiac disorders who have a reduced hepatic tolerance to intra- and postoperative hemodynamic changes. Our laboratory findings support this hypothesis, as alkaline phosphatase and bilirubin are expected to be increased while transaminases are often normal or only slightly elevated in patients with cardiac hepatopathy as long as cardiac output is not severely compromised [28, 33].

The present study population was exposed to severe hemodynamic fluctuations due to low cardiac output on one hand [34, 35] and ECMO support itself on the other [36–38]. ECMO therapy may affect liver perfusion due to changes in pump flow and the lack of pulsatility, rapid changes of vasopressors doses after ECMO initiation, and air or thrombus embolization [36–38]. This hemodynamic instability in ECMO patients may explain the pronounced impact of bilirubin and alkaline phosphatase on clinical outcome. Although, in agreement with the current literature [32], hypoxic hepatitis was found to be strongly associated with mortality in the present study population, no association of preoperative liver function parameters with the occurrence of hypoxic hepatitis could be shown. In addition to these parameters, low albumin plasma levels, which indicate an impairment of synthetic liver function, were found to be associated with poor long-term survival. This finding is in line with previous data identifying albumin levels as predictive of mortality in critically ill patients [39]. Albumin reflects the burden of noncardiac comorbidities, as it is reduced in patients with diabetes mellitus, chronic kidney disease, and severe chronic obstructive pulmonary disease [40]. This association may explain the association with long-term but not short-term mortality.

Some potential limitations of the present study need to be considered. First, although the presented number of patients and the median follow-up are among the highest in the current literature, they might still not be sufficient to draw definitive conclusions. Second, the generalizability of our results might be limited, as our study reflects the experience at a single center and the population was highly specific, with a significant proportion of patients undergoing heart transplantation and ventricular assist device implantation. However, in view of the dramatic increase in the number of ventricular assist device implants [41], this specific population has become increasingly relevant over the last several years. Third, the ECMO patients under investigation represent a specific study population, and it remains to be tested whether our findings apply exclusively to this highly vulnerable patient cohort or could be transferred to a general population of surgical patients.

## Conclusions

The present study demonstrates that elevated values of alkaline phosphatase and total bilirubin are sensitive parameters for predicting the short-term and long-term outcomes of ECMO patients. Our findings extend the limited information on risk stratification in patients undergoing ECMO support and represent a valuable and easily available addition incomprehensive decision-making before ECMO implantation.

## Key messages

- ECMO represents a valuable and rapidly evolving therapeutic option in patients with severe heart or lung failure following cardiovascular surgery.
- Alkaline phosphatase and total bilirubin are sensitive parameters for predicting short-term and long-term outcomes in patients undergoing ECMO support.
- Alkaline phosphatase and total bilirubin represent a valuable and easily available addition for comprehensive decision-making before ECMO implantation.

## Additional file

Additional file 1: Table S1. Baseline characteristics of ECMO study population by type of cardiovascular surgery. **Table S2** Baseline characteristics of ECMO study population by time of study inclusion. (DOCX 27 kb)

## Abbreviations

ALT: alanine aminotransferase
AST: aspartate aminotransferase
CABG: coronary artery bypass graft
CI: confidence interval
ECMO: extracorporeal membrane oxygenation
eGFR: estimated glomerular filtration rate
EuroSCORE: European System for Cardiac Operative Risk Evaluation
FiO_2_: fraction of inspired oxygen
HR: hazard ratio
ICU: intensive care unit
IQR: interquartile range
SAPS: Simplified Acute Physiology Score
ScVO_2_: central venous oxygen saturation
SD: standard deviation
SOFA: Sequential Organ Failure Assessment
