# Supplementary material for: Liver function predicts survival in patients undergoing extracorporeal membrane oxygenation following cardiovascular surgery
Source: Crit Care. 2016 Mar 11;20:57. doi: 10.1186/s13054-016-1242-4 (PMC4788876; doi:10.1186/s13054-016-1242-4)
Supplement: Additional file 1: Table S1. — Baseline characteristics of ECMO study population by type of cardiovascular surgery. Table S2 Baseline characteristics of ECMO study population by time of study inclusion. (DOCX 27 kb) [file 13054_2016_1242_MOESM1_ESM.docx]

**Table S1:** Baseline characteristics of ECMO study population by type of cardiovascular surgery.

|  | **CABG**  **(n=24)** | **Valve ±CABG**  **(n=115)** | **HTX**  **(n=51)** | **VAD**  **(n=21)** | **Aortic reconst.**  **(n=17)** | **Other CV surgery**  **(n=12)** |
| --- | --- | --- | --- | --- | --- | --- |
| **Baseline characteristics at hospital admission** | | |  |  |  |  |
| Age, median years (IQR) | 70 (60- 76) | 70 (62- 77) | 58 (52- 65) | 57 (47- 67) | 59 (41- 66) | 58 (47- 64) |
| Male sex n (%) | 17 (71) | 74 (64) | 44 (86) | 19 (90) | 12 (71) | 6 (50) |
| EuroSCORE (additive), points (IQR) | 11 (8- 13) | 10 (8- 13) | 9 (7- 12) | 11 (10- 13) | 12 (9- 13) | 10 (5-12) |
| Procedure duration, h:min (IQR) | 7:25 (4:15- 9:10) | 8:10 (6:08- 9:37) | 8:30 (6:50- 10:25) | 6:25 (6:05- 8:23) | 8:07 (6:30- 9:18) | 7:05 (5:10- 9:20) |
| Hypertension, n (%) | 18 (75) | 87 (76) | 30 (59) | 12 (57) | 15 (88) | 7 (58) |
| Diabetes, n (%) | 9 (38) | 35 (30) | 11 (22) | 9 (43) | 0 (0) | 2 (17) |
| Hypercholesterolemia, n (%) | 15 (63) | 60 (52) | 23 (45) | 13 (62) | 10 (59) | 4 (33) |
| Coronary artery disease, n (%) | 17 (71) | 65 (57) | 19 (37) | 11 (52) | 7 (41) | 5 (42) |
| Left ventricular ejection fraction |  |  |  |  |  |  |
| 30-44%, n (%) | 5 (21) | 20 (17) | 5 (10) | 0 (0) | 2 (12) | 3 (25) |
| <30%, n (%) | 7 (29) | 25 (22) | 39 (76) | 16 (76) | 0 (0) | 7 (58) |
| Creatinine, mg/dl (IQR) | 1.5 (1.2- 2.0) | 1.3 (1.0- 1.8) | 1.6 (1.2- 2.1) | 1.4 (1.2- 1.9) | 1.2 (1.0- 1.4) | 1.3 (0.9- 1.6) |
| Estimated GFR, ml/min/1.73 m^2^ (IQR) | 48.1 (31.4- 67.0) | 51.6 (39.4- 68.5) | 46.5 (31.7- 68.2) | 52.8 (35.7- 66.8) | 63.7 (56.5- 73.0) | 48.9 (38.9- 83.8) |
| Blood urea nitrogen, mg/dL (IQR) | 23.8 (18.9- 36.1) | 24.1 (18.2- 37.9) | 28.4 (18.9- 50.2) | 27.3 (19.0- 44.5) | 18.7 (15.1- 22.7) | 21.5 (16.5- 31.5) |
| Cholesterol, mg/dl (IQR) | 143 (96- 172) | 144 (104- 183) | 145 (112- 194) | 91 (72- 127) | 174 (143- 205) | 137 (91- 175) |
| C-reactive protein, mg/dl (IQR) | 0.9 (0.2- 6.4) | 1.0 (0.3- 4.3) | 0.9 (0.4- 2.0) | 3.5 (1.2- 8.7) | 0.4 (0.2- 1.2) | 1.9 (0.6- 10.8) |
| White blood cell, G/l (IQR) | 7.9 (6.0- 12.1) | 7.7 (6.2- 10.4) | 7.7 (5.9- 10.4) | 9.0 (7.7- 11.1) | 9.1 (5.9- 16.5) | 8.3 (6.2- 14.1) |
| Thrombocyte, G/l (IQR) | 231 (171- 252) | 175 (123- 232) | 190 (143- 241) | 172 (122- 232) | 200 (166- 240) | 200 (90- 260) |
| **Liver parameters** | | |  |  |  |  |
| Bilirubin total, mg/dl (IQR) | 0.7 (0.5- 1.1) | 1.1 (0.6- 1.8) | 1.0 (0.6- 1.6) | 1.4 (1.1- 2.2) | 0.7 (0.6- 1.0) | 0.7 (0.5- 1.8) |
| Alkaline phosphatase, U/l (IQR) | 81 (50- 101) | 75 (61- 99) | 104 (74- 161) | 121 (68- 173) | 67 (58- 83) | 58 (55- 88) |
| Aspartate transaminase, U/l (IQR) | 31 (21- 150) | 30 (23- 53) | 35 (26- 68) | 47 (25- 201) | 39 (27- 59) | 69 (24- 511) |
| Alanine transaminase, U/l (IQR) | 27 (18- 40) | 25 (16- 34) | 34 (19- 54) | 30 (25- 192) | 36 (20- 63) | 32 (18- 58) |
| AST/ALT ratio, n (IQR) | 1.3 (0.9- 2.1) | 1.3 (1.0- 2.3) | 1.1 (0.8- 1.8) | 1.1 (1.0- 1.8) | 1.1 (0.9- 1.6) | 1.8 (1.3- 6.0) |
| Gamma-glutamyltransferase, U/l (IQR) | 47 (29- 111) | 48 (26- 85) | 103 (43- 190) | 120 (53- 258) | 45 (30- 55) | 38 (22- 68) |
| Albumin, g/l (IQR) | 38.2 (34.2- 43.3) | 37.6 (28.4- 42.2) | 40.5 (36.5- 44.1) | 32.3 (25.1- 39.5) | 40.5 (38.0- 43.2) | 30.5 (26.3- 40.5) |
| Normotest, % (IQR) | 88 (80- 102) | 77 (59- 98) | 43 (24- 75) | 61 (39- 84) | 82 (74- 100) | 85 (49- 106) |
| **Post ECMO implantation (first 24hours)** | | |  |  |  |  |
| SAPS III, n (%) | 45 (36- 51) | 43 (37- 54) | 44 (38- 48) | 40 (34- 50) | 40 (36- 51) | 45 (30- 58) |
| SOFA score, n (IQR) | 12 (11- 13) | 12 (11- 14) | 12 (10- 15) | 10 (9- 13) | 13 (11- 14) | 13 (10- 13) |
| ECMO flow, l/min (IQR) | 3.05 (2.60- 4.09) | 3.48 (2.77- 4.20) | 4.00 (2.90- 5.00) | 2.10 (1.95- 3.00) | 3.28 (2.60- 4.00) | 2.66 (2.50- 3.20) |
| ECMO rotation, rpm (IQR) | 3155 (2400- 3545) | 2970 (2580- 3410) | 3500 (3000- 3800) | 2330 (2060- 2910) | 2590 (2310- 3200) | 3110 (2460- 3700) |
| ECMO gas flow, l/min (IQR) | 2.5 (1.8- 3.0) | 2.5 (2.0- 3.0) | 2.8 (2.0- 3.0) | 2.8 (2.0- 4.0) | 3.0 (2.0- 3.0) | 2.5 (2.0- 3.0) |
| ECMO FiO2, % (IQR) | 67 (60- 80) | 60 (60- 80) | 60 (60- 70) | 100 (70- 100) | 95 (70- 100) | 80 (70- 100) |
| ECMO duration, median days (IQR) | 5 (3- 6) | 5 (3- 7) | 4 (3- 7) | 2 (2- 4) | 4 (3- 7) | 2 (2- 7) |
| **Hemodynamic parameters (at ICU admission)** | | |  |  |  |  |
| Mean arterial pressure, mmHg (IQR) | 74 (67- 79) | 70 (63- 78) | 73 (66- 80) | 71 (66- 76) | 75 (69- 78) | 76 (67- 78) |
| Cardiac output, l/min | 4.0 (3.2- 4.8) | 3.6 (2.8- 5.1) | 4.0 (2.8- 5.1) | 5.1 (3.7- 6.0) | 1.8 (1.1- 2.4) | 3.6 (3.1- 6.0) |
| ScVO2, % (IQR) | 73 (66-76) | 69 (63- 77) | 71 (62- 78) | 71 (67- 78) | 75 (71- 76) | 72 (59- 72) |
| Central venous pressure, mmHg (IQR) | 14 (12- 16) | 14 (11- 17) | 14 (10-16) | 14 (13- 16) | 13 (12- 15) | 14 (12- 17) |
| **Medication (first 24hours post ECMO)** | | |  |  |  |  |
| Noradrenaline, n (%) | 23 (96) | 113 (98) | 50 (98) | 19 (90) | 17 (100) | 12 (100) |
| Noradrenaline (max. dose), μg/kg/min | 0.25 (0.12- 0.69) | 0.36 (0.18- 0.65) | 0.23 (0.12- 0.48) | 0.17 (0.08- 0.24) | 0.19 (0.13- 0.48) | 0.34 (0.23- 0.54) |
| Dobutamine, n (%) | 24 (100) | 102(89) | 50 (98) | 18 (86) | 13(76) | 11 (92) |
| Dobutamine (max. dose), μg/kg/min | 4.28 (2.46- 8.29) | 4.80 (3.09- 6.61) | 5.56 (3.70- 9.26) | 4.56 (2.92- 7.25) | 2.78 (1.18- 3.70) | 4.88 (2.42- 6.67) |
| Vasopressin, n (%) | 7 (29) | 42 (37) | 17 (33) | 7 (33) | 6 (35) | 6 (50) |
| Vasopressin (max. dose), U/h (IQR) | 3.5 (3.0- 5.0) | 3.0 (2.0- 4.0) | 4.0 (2.5- 4.0) | 2.0 (1.5- 3.0) | 2.0 (2.0- 3.0) | 3.0 (2.0- 3.0) |

**Table S2:** Baseline characteristics of ECMO study population by time of study inclusion.

|  | **Patients included**  **2003- 2008 (n=76)** | **Patients included**  **2009- 2014 (n=164)** |
| --- | --- | --- |
| **Baseline characteristics at hospital admission** | | |
| Age, median years (IQR) | 65 (56-71) | 65 (55- 73) |
| Male sex n (%) | 56 (74) | 116 (71) |
| EuroSCORE (additive), points (IQR) | 9 (7- 12) | 11 (8- 13) |
| Procedure duration, h:min (IQR) | 8:27 (6:00- 9:25) | 7:39 (6:10- 10:00) |
| Hypertension, n (%) | 39 (51) | 130 (79) |
| Diabetes, n (%) | 21 (28) | 45 (27) |
| Hypercholesterolemia, n (%) | 24 (32) | 101 (62) |
| Coronary artery disease, n (%) | 38 (50) | 86 (52) |
| Left ventricular ejection fraction |  |  |
| 30-44%, n (%) | 15 (20) | 20 (12) |
| <30%, n (%) | 31 (41) | 63 (38) |
| Creatinine, mg/dl (IQR) | 1.3 (1.1- 1.8) | 1.3 (1.0- 1.8) |
| Estimated GFR, ml/min/1.73 m^2^ (IQR) | 50.9 (39.3- 66.5) | 51.5 (38.4- 70.4) |
| Blood urea nitrogen, mg/dL (IQR) | 25.3 (19.5- 37.9) | 23.2 (17.3- 36.1) |
| Cholesterol, mg/dl (IQR) | 139 (99- 185) | 144 (106- 179) |
| C-reactive protein, mg/dl (IQR) | 1.5 (0.5- 8.3) | 0.9 (0.3- 3.5) |
| White blood cell, G/l (IQR) | 8.8 (6.5- 13.3) | 7.8 (5.9- 10.2) |
| Thrombocyte, G/l (IQR) | 175 (120- 239) | 190 (141- 244) |
| **Liver parameters** | | |
| Bilirubin total, mg/dl (IQR) | 1.1 (0.7- 1.8) | 0.9 (0.6- 1.5) |
| Alkaline phosphatase, U/l (IQR) | 80 (57- 110) | 81 (63- 109) |
| Aspartate transaminase, U/l (IQR) | 37 (25- 97) | 31 (23- 55) |
| Alanine transaminase, U/l (IQR) | 27 (17- 47) | 26 (18- 49) |
| AST/ALT ratio, n (IQR) | 1.5 (1.0- 2.9) | 1.2 (0.9- 1.7) |
| Gamma-glutamyltransferase, U/l (IQR) | 56 (36- 111) | 52 (31- 105) |
| Albumin, g/l (IQR) | 37 (26- 43) | 39 (32- 42) |
| Normotest, % (IQR) | 72 (47- 91) | 77 (56- 96) |
| **Post ECMO implantation (first 24hours)** | | |
| SAPS III, n (%) | 43 (37- 50) | 43 (36- 53) |
| SOFA score, n (IQR) | 12 (10- 14) | 12 (10- 14) |
| ECMO flow, l/min (IQR) | 4.13 (3.25- 4.95) | 3.20 (2.50- 4.09) |
| ECMO rotation, rpm (IQR) | 3500 (3000- 3900) | 2960 (2440- 3410) |
| ECMO gas flow, l/min (IQR) | 3.0 (3.0- 4.0) | 2.5 (2.0- 3.0) |
| ECMO FiO2, % (IQR) | 60 (50- 80) | 70 (60-100) |
| ECMO duration, median days (IQR) | 4 (2- 6) | 5 (3- 7) |
| **Hemodynamic parameters (at ICU admission)** | | |
| Mean arterial pressure, mmHg (IQR) | 73 (65- 80) | 72 (65- 78) |
| Cardiac output, l/min | 3.7 (2.9- 5.2) | 3.9 (2.8- 5.1) |
| ScVO2, % (IQR) | 72 (64- 76) | 71 (65- 77) |
| Central venous pressure, mmHg (IQR) | 14 (12- 17) | 13 (11- 16) |
| **Medication (first 24hours post ECMO)** | | |
| Noradrenaline, n (%) | 72 (95) | 162 (99) |
| Noradrenaline (max. dose), μg/kg/min | 0.30 (0.12- 0.62) | 0.28 (0.14- 0.53) |
| Dobutamine, n (%) | 66 (87) | 152 (93) |
| Dobutamine (max. dose), μg/kg/min | 5.32 (3.29- 8.25) | 4.44 (2.78- 6.81) |
| Vasopressin, n (%) | 9 (12) | 76 (46) |
| Vasopressin (max. dose), U/h (IQR) | 4.0 (2.0- 4.0) | 3.0 (2.0- 4.0) |
